# Solasodine suppresses nasopharyngeal carcinoma progression by inducing ferroptosis

**DOI:** 10.1038/s41598-025-93834-4

**Published:** 2025-05-18

**Authors:** Jing Wang, DongHua Wang, SiQing Ma, BinSheng He, JiaoYang Lu, Zhen Guo

**Affiliations:** 1 Hunan Provincial Key Laboratory of the Traditional Chinese Medicine Agricultural Biogenomics, Changsha Medical University, Changsha, 410219 China; 2 Hunan Provincial Key Laboratory of the Fundamental and Clinical Research on Functional Nucleic Acid, Changsha Medical University, Changsha, 410219 China; 3 The First Clinical College, Changsha Medical University, Changsha, 410219 China; 4 School of Nursing, Changsha Medical University, Changsha, 410219 China; 5 Department of Pharmacy, Hunan Chest Hospital, Changsha, 410013 China; 6 Hunan Provincial Key Laboratory of the Research and Development of Novel Pharmaceutical Preparations, Changsha Medical University, Changsha, 410219 China

**Keywords:** Nasopharyngeal carcinoma, Solasodine, Ferroptosis, Network pharmacology, Cell death, Biochemistry, Cancer, Cell biology

## Abstract

Nasopharyngeal carcinoma (NPC) is a malignant tumor with a high prevalence in China. Solasodine is a natural compound derived from the traditional herb that possess anticancer activity in various tumors, but its role in NPC remains unclear. Here, we demonstrated that solasodine potently suppressed NPC growth and induced cell death both in vitro and in vivo. Network pharmacology identified HMOX1 as a pivotal target of solasodine linked to ferroptosis. Solasodine triggered ferroptotic hallmarks, including mitochondrial cristae disruption, elevated Fe^2^⁺/ROS/MDA, depleted GSH, and dysregulated ferroptosis-related proteins (HMOX1/COX2↑, GPX4/MUC1/SLC40A1↓). Crucially, ferroptosis inhibitors (Fer-1/Lip-1), but not apoptosis, necroptosis, or autophagy inhibitors, rescued solasodine-induced cell death, confirming ferroptosis as the dominant mechanism. In conclusion, by applying network pharmacology accompanied with experimental validation, our study unveils solasodine as a novel ferroptosis inducer for NPC treatment. However, its therapeutic potential requires further validation in patient-derived models and clinical trials.

## Introduction

Nasopharyngeal carcinoma (NPC) is a malignant tumor with significant geographic distribution difference and is highly prevalent in southern China^1^. Radiotherapy and chemotherapy are the main treatments for NPC. Although early-stage patients can be cured by radiotherapy alone, advanced-stage patients are still prone to treatment-resistant reactions such as metastasis and recurrence after chemoradiotherapy, which leads to treatment failure^2^. Therefore, it is of great significance to find new effective therapeutic targets and drugs for NPC to improve the prognosis of NPC patients.

The development of novel anticancer drugs from natural plants has been a hot topic in tumor therapy^3,4^. Natural compounds such as allicin, berberine and luteolin have been reported to induce ferroptosis in NPC cells, leading to reduced cell proliferation, metastasis and increased oxidative stress^5–7^. Solasodine is a steroidal alkaloid isolated from *Solanum incanum L.*, which has a variety of pharmacological activities such as antitumor, antibacterial, anti-inflammatory, antioxidant, etc.^8,9^. In recent years, it has been found that solasodine has an inhibitory effect on a variety of cancers including lung cancer, gastric cancer, hepatocellular carcinoma, colorectal cancer, pancreatic cancer, breast cancer, which has a great potential in cancer treatment^10^. For example, solasodine suppresses AKT/GSK-3b/b-catenin signaling and reverses epithelial-mesenchymal transition, thereby exerting an anti-cancer effect in colorectal cancer^11,12^. Solasodine can directly suppresses Hh/Gli1 signaling and target cancer stem-like cells (CSCs) in breast cancer^13^. Solasodine inhibits the metastasis of gastric cancer through regulating the AMPK/STAT3/NF-κB pathway^14^. Solasodine induces autophagy and inhibits metastasis in ovarian cancer^15^.

Notably, the steroidal backbone and pro-oxidant effects of solasodine across cancers suggest an unexplored capacity to disrupt redox homeostasis—a hallmark of ferroptosis. Ferroptosis is a distinct form of cell death that is characterized by iron-dependent lipid peroxidation and accumulation of reactive oxygen species^16,17^. This process is tightly regulated and involves various metabolic pathways, including iron metabolism, lipid metabolism, and the glutathione (GSH) antioxidant system, particularly the enzyme glutathione peroxidase 4 (GPX4)^18^. Notably, emerging evidence indicates that NPC cells exhibit heightened susceptibility to ferroptosis due to dysregulated iron metabolism and altered lipid peroxidation defenses. Clinical studies reveal that NPC patients exhibit functional iron deficiency (low serum iron and transferrin levels), creating a unique metabolic vulnerability that could be exploited by ferroptosis-inducing agents^19^. This pro-oxidant potential of solasodine aligns with NPC’s unique iron metabolism dysregulation, making ferroptosis a promising therapeutic avenue.

Recent studies have highlighted the role of ferroptosis as a potential therapeutic target in NPC. Studies indicate that NPC cells exhibit a unique sensitivity to ferroptosis inducers, which can be leveraged to counteract chemoresistance. For instance, the induction of ferroptosis has been shown to inhibit the proliferation and survival of NPC cells, thereby enhancing the efficacy of chemotherapeutic agents like cisplatin, sorafenib and gemcitabine^20–22^. Research demonstrates that agents such as dihydroartemisinin (DHA) can synergistically enhance the cytotoxic effects of cisplatin in resistant NPC cell lines by promoting ferroptosis^23^. Radiotherapy in conjunction with ferroptosis inducers has been shown to amplify the therapeutic effects in NPC by repressing SLC7A11, which is critical for cystine uptake and glutathione synthesis, thereby promoting lipid peroxidation and ferroptosis^24,25^. Moreover, the use of nanomedicine strategies, such as iron oxide nanoparticles combined with radiotherapy, has demonstrated significant potential in enhancing ferroptosis and improving the radiosensitivity of NPC cells^26^. Therefore, the concurrent targeting of ferroptosis during radiotherapy and chemotherapy represents a novel and effective strategy to enhance treatment outcomes in patients with NPC.

Based on the chemoresistance challenges in advanced NPC and the therapeutic potential of ferroptosis induction, we hypothesized that solasodine inhibits NPC progression by triggering ferroptosis. To test this, we combined in vitro/in vivo functional assays with network pharmacology to map solasodine’s targets and focused on the ferroptosis regulator HMOX1. We further validated ferroptotic events through metabolomic and morphological analyses, aiming to establish solasodine as a novel therapeutic candidate for NPC.

## Methods and materials

### Cell culture

NPC cells (HONE1 and HNE1) were obtained from the Cell Bank of Central South University (Changsha, China) with STR authentication and cultivated in RPMI-1640 Medium (PM150110, Pricella Biotechnology) supplemented with penicillin (100 U/mL), streptomycin (100 μg/mL), and 10% fetal bovine serum (FBSST-01033-500, Cyagen Biosciences) at 37 °C in a humidifed atmosphere of 5% CO_2_.

### Reagents and antibodies

The reagents used in this study include solasodine (SD) (AB1661, Chengdu Alfa Biotechnology), Liproxstatin-1 (50 nM; HY-12726, MCE), Ferrostatin-1 ( 1 μM; M2698, Abmole), Necrostatin-1 (10 μM; N860905, Macklin), Z-VAD-FMK (10 μM; Z860402, Macklin), 3-MA (60 μM; M833793, Macklin), DCFH-DA (S0033S, Beyotime), C11-BODIPY581/591 (D3861, Invitrogen), DMSO (ST2335, Beyotime). The antibodies used in this study include GPX4 (381958, Zenbio), HMOX1 (AF5393, Affinity), COX2 (T58852, Abmart), SLC40A1 (TD13561, Abmart), MUC1 (PK41366S, Abmart), β-Actin (AC050, ABclonal), HRP Goat Anti-Rabbit IgG (H + L) antibody (AS014, ABclonal), Ki67 (27309-1-AP, Proteintech).

### Cell viability assay

Cell viability was assessed using Cell Counting Kit-8 (GK10001, GlpBio) in accordance with the manufacturer’s instructions. NPC cells (HONE1 and HNE1) were seeded in 96-well flat bottom microtiter plates. After SD treatment for the indicated durations, cells were rinsed twice with phosphate-buffered saline (PBS). Then, 100 μL of medium containing 10 μL of CCK8 solution was added to cells and incubated at 37 °C for 1.5 h. Absorbance was measured at a wavelength of 450 nm using the microplate reader (Synergy H11, BioTek).

### Cell cycle analysis

After SD treatment for 48 h, NPC cells (HONE1 and HNE1) were harvested for cell cycle analysis. The fixed cells were rinsed with PBS twice followed by staining with 150 μL of propidium iodide (PI) working solution (MB2920, MeilunBio) in the dark at 4 °C for 30 min to label the DNA. The stained cells were transferred to flow cytometry tubes and analyzed on a Flow Cytometer (FACSCelesta, BD). A total of 10,000 cells were acquired based on forward scatter (FSC) and side scatter (SSC) dot plots. Gating techniques were applied to exclude doublets and debris. The percentage of cells in each phase of the cell cycle was quantified from the PI fluorescence histogram using FlowJo V10 software.

### Colony formation assay

A colony formation assay was conducted to evaluate the ability of NPC cells (HONE1 and HNE1) to form colonies. Cells were plated in 6-well plates at a density of 1000 cells per well and cultured for two weeks. Colonies were then fixed with 4% paraformaldehyde and stained with 0.1% crystal violet at room temperature for 30 min. After staining, colonies were imaged and counted.

### Cell death assessment

Annexin V-FITC Apoptosis Detection Kit (C1062L, Beyotime) was used to assess apoptosis in NPC cells following SD treatment. All cells including those detached into the culture medium were collected and gently resuspended in ice-cold 1 × binding buffer at a concentration of 1 × 10^6^ cells/ml. 100 μL of cell suspension were mixed with 5 μL FITC Annexin V and 5 μL PI, followed by incubation for 15 min at room temperature in the dark. The apoptotic cell fraction was subsequently quantified using a flow cytometry. Additionally, to visualize live and dead cells, NPC cells in 24-well plates were treated with SD for specified durations and then stained with 100 nM SYTO-9 Green (S34854, Invitrogen) and 20 μg/mL PI. After a 20-min incubation in the dark at room temperature, the stained cells were rinsed with PBS three times and then examined under an automated fluorescent inverted microscope (EVOS M7000, Thermo). The quantification of live and dead cells was achieved using ImageJ software.

### Xenograft tumor model

The animal experiment was approved by the Animal Research Ethics Committee of Changsha Medical University (No. 2023049) and carried out in accordance with relevant guidelines and regulations. All animal experiment procedures and methods were reported in accordance with ARRIVE guidelines. The HNE1 cells (1 × 10^7^ cells in 0.1 ml PBS) were subcutaneously injected into the armpit of 5-week-old female BALB/c nude mice (SLAC Laboratory Animal Center, Shanghai, China). When the tumor volume reaches approximately 150 mm^3^ (around 7 days post-injection), the mice were randomly divided into two groups (5 mice/group): (i) the control group received methanol; (ii) the intervention group received solasodine (dissolved in methanol, 10 mg/kg body weight). Solasodine were administered by intraperitoneal injection every three days for a total of 7 times. Tumor volumes were measured twice a week and calculated as 0.5 × length × width^2^. After 3 weeks of drug administration, the mice were anesthetized with carbon dioxide and euthanized by cervical dislocation. The tumors were resected and subjected to TUNEL staining assay and immunohistochemical experiments.

### Immunohistochemistry

Xenograft tumor tissue specimens were initially fixed in 10% formalin, paraffin-embedded, and sectioned into 4 μm slices. For immunohistochemical staining, paraffin-embedded sections were subjected to dewaxing and rehydration in a series of graded ethanol baths followed by distilled water rinses. Antigen retrieval involved heat treatment in citrate buffer, preventing buffer evaporation, and subsequent cooling with PBS washes on a shaker for 5 min. Endogenous peroxidase inhibition was achieved with 3% H₂O₂ for 25 min at room temperature, followed by PBS washes. Tissue sections were blocked with 3% BSA for 30 min at room temperature. The primary antibody was incubated overnight at 4 °C in a humidified chamber after removing the blocking serum. An HRP-conjugated secondary antibody was applied and incubated for 50 min at room temperature. Nuclei were counterstained with hematoxylin, differentiated, and returned to a blue color. Dehydration proceeded through increasing ethanol concentrations, clearing in xylene, and mounting with coverslips. Immunohistochemical images were finally evaluated using a pathological section scanner (Pannoramic MIDI, 3DHISTECH).

### TUNEL staining

Xenograft tumor tissues were processed through fixation, dehydration, paraffin embedding, and sectioning. To quantify apoptosis, terminal deoxynucleotidyl transferase dUTP nick end labeling (TUNEL) staining was conducted using a TUNEL Kit (C1089, Beyotime) in accordance with the manufacturer’s protocol. Preparatory steps included dewaxing in xylene for 5–10 min, followed by two cycles of fresh xylene and graded ethanol dehydration. Tissues were then treated with DNase-free proteinase K (20 μg/mL) at 37 °C for 15–30 min. Thorough washing with PBS was essential to remove residual proteinase K. The TUNEL reaction was initiated by adding 50 μL of TUNEL reaction mixture to the samples, followed by incubation at 37 °C for 60 min in the dark. Post-reaction washing with PBS was conducted before mounting with antifade mounting medium for fluorescence microscopy observation. The apoptotic index was determined by calculating the proportion of TUNEL-positive nuclei relative to the total nuclei, as identified by 4’,6-diamidino-2-phenylindole (DAPI) staining.

### Network pharmacology

The corresponding 2D and 3D structures of solasodine (Compound CID: 442985) were downloaded from the Pubchem database (https://pubchem.ncbi.nlm.nih.gov/). The PharmMapper database (http://www.lilab-ecust.cn/pharmmapper/submitfle.html) was used to obtain the predicted gene targets of solasodine. Then, the differentially expressed genes (DEGs) of NPC cells treated with solasodine or solvent control were obtained. Intersection genes between the predicted targets of solasodine and DEGs of NPC cells treated with solasodine were produced using Venn diagrams (https://bioinfogp.cnb.csic.es/tools/venny/) and imported into the STRING database (https://bioinfogp.cnb.csic.es/tools/venny/) to obtain a protein–protein interaction (PPI) network. The obtained PPI data were imported into Cytoscape3.9.1 for analysis and construction of the hub genes. Metascape (https://metascape.org/gp/index.html#/main/step1) was utilized to perform GO and KEGG pathway enrichment analyses. GO analysis revealed significant enrichment of genes in biological process (BP), cellular component (CC), and molecular function (MF) categories.

### Transmission electron microscopy

After a 48-h exposure to SD, NPC cells were enzymatically dissociated using trypsin, centrifuged to obtain cell pellets, and promptly preserved at 4 °C in a fixative comprising 2.5% glutaraldehyde. Subsequent to postfixation with 1% osmium tetroxide, the cells underwent progressive dehydration through a series of graded ethanol solutions. Once adequately dehydrated, the samples were embedded in epoxy resin and sliced into approximately 50 nm sections for observation. The sections which stained with lead citrate and uranyl acetate were observed using a transmission electron microscope (TEM) (JEM-1400, JEOL), enabling the visualization of intricate cellular morphology and the detailed organization of subcellular structures.

### MitoTracker Red CMXRos staining

NPC cells were seeded into 24-well plates and permitted to adhere and proliferate for 24 h. Subsequently, the cells received a 24-h treatment with 32 μM SD and then incubated with MitoTracker Red CMXRos (C1035, Beyotime) following the manufacturer’s instructions. Post-incubation, cells were rinsed with phosphate-buffered saline (PBS) to remove excess dye, and then imaged using an automated fluorescent inverted microscope. The mitochondrial status was quantitatively assessed using ImageJ software.

### Glutathione (GSH) assay

The intracellular concentration of reduced GSH was quantified using the Reduced Glutathione (GSH) Colorimetric Assay Kit (E-BC-K030-M, Elabscience) according to the manufacturer’s protocol. The absorbance of the assay solution was measured at 405 nm using a microplate reader. The GSH content in the cellular lysates was then determined by applying the calculation formula specified in the assay kit’s product documentation.

### Iron detection

The concentration of intracellular ferrous iron (Fe^2+^) was evaluated using the Cell Ferrous Iron Colorimetric Assay Kit (E-BC-K881-M, Elabscience), following the manufacturer’s detailed procedure. The absorbance of the assay solution was measured at 593 nm using a microplate reader. The Fe^2+^ content within the cellular extracts was subsequently computed utilizing the specific equation outlined in the assay kit’s accompanying protocol.

### Malondialdehyde (MDA) assay

The concentration of MDA, a marker of lipid peroxidation, was quantified using the Malondialdehyde (MDA) Colorimetric Assay Kit (E-BC-K028-M, Elabscience) according to the manufacturer’s instructions. The assay solution’s absorbance was measured at 532 nm using a microplate reader. The MDA content present in the cellular lysates was then determined by applying the calculation formula as delineated in the assay kit’s product protocol.

### Reactive oxygen species (ROS) assay

For the detection of intracellular ROS levels, cells were incubated with the DCFH-DA fluorescent probe at a final concentration of 10 μM in serum-free medium at 37 °C for 30 min. After the incubation, cells were thoroughly washed three times with PBS to remove any unbound probe. The green fluorescence, served as a direct indicator of ROS production, was subsequently visualized using an automated fluorescent inverted microscope and quantitatively assessed using ImageJ software.

### Lipid peroxidation assay

For the assessment of lipid peroxidation, cells were incubated in PBS supplemented with 5 μM C11-BODIPY581/591 for 30 min at 37 °C under dark conditions. The probe exhibits a distinct fluorescence emission peak shift from approximately 590 to 510 nm, reflecting the degree of lipid peroxidation. This spectral alteration was captured through the use of a Flow Cytometer, which facilitated the collection of fluorescence data. The subsequent analysis of these data was conducted using the FlowJo V10 software.

### Western blot

Proteins were harvested from cells using RIPA buffer (P0013C, Beyotime) supplied with PMSF, and their concentrations were determined via the BCA Protein Assay Kit (P0012S, Beyotime) following the manufacturer’s guidelines. An optimal protein load of 30–50 μg per lane was subjected to SDS-PAGE and electroblotted onto PVDF membranes. Non-specific binding sites on the membranes were blocked with 5% non-fat milk in TBST (Tris-buffered saline with Tween 20) at room temperature for 2 h. Subsequently, the membranes were incubated overnight at 4 °C with primary antibodies respectively, including rabbit anti-GPX4 (1:1000), rabbit anti-HMOX1 (1:1000), rabbit anti-COX2 (1:1000), rabbit anti-SLC40A1 (1:1000), rabbit-MUC1 (1:1000), and rabbit anti-β-actin (1:10,000). Following this, the membranes were washed three times with TBST, and further incubated with HRP Goat Anti-Rabbit IgG (H + L) antibody (1:10,000) for 1.5 h at room temperature. After washing three times with TBST, the membranes were detected using the BeyoECL Star Ultra Hyperensitive ECL Chemiluminescence Kit (P0018AS, Beyotime).

### Statistical analysis

Statistical analysis was performed with GraphPad Prism 6.0 software (SanDiego, USA). A T-test was used to compare two independent groups, while oneway ANOVA was used to evaluate differences among groups. Unpaired *t* test with Welch’s correction was used to assess whether non-normally distributed data were significantly different. A probability (*p*) value of < 0.05 was considered statistically significant. All results are presented as the mean ± standard deviation (SD). All data were collected from at least three independent experiments.

## Results

### Solasodine inhibits the viability and proliferation of NPC cells

**Fig. 1:**
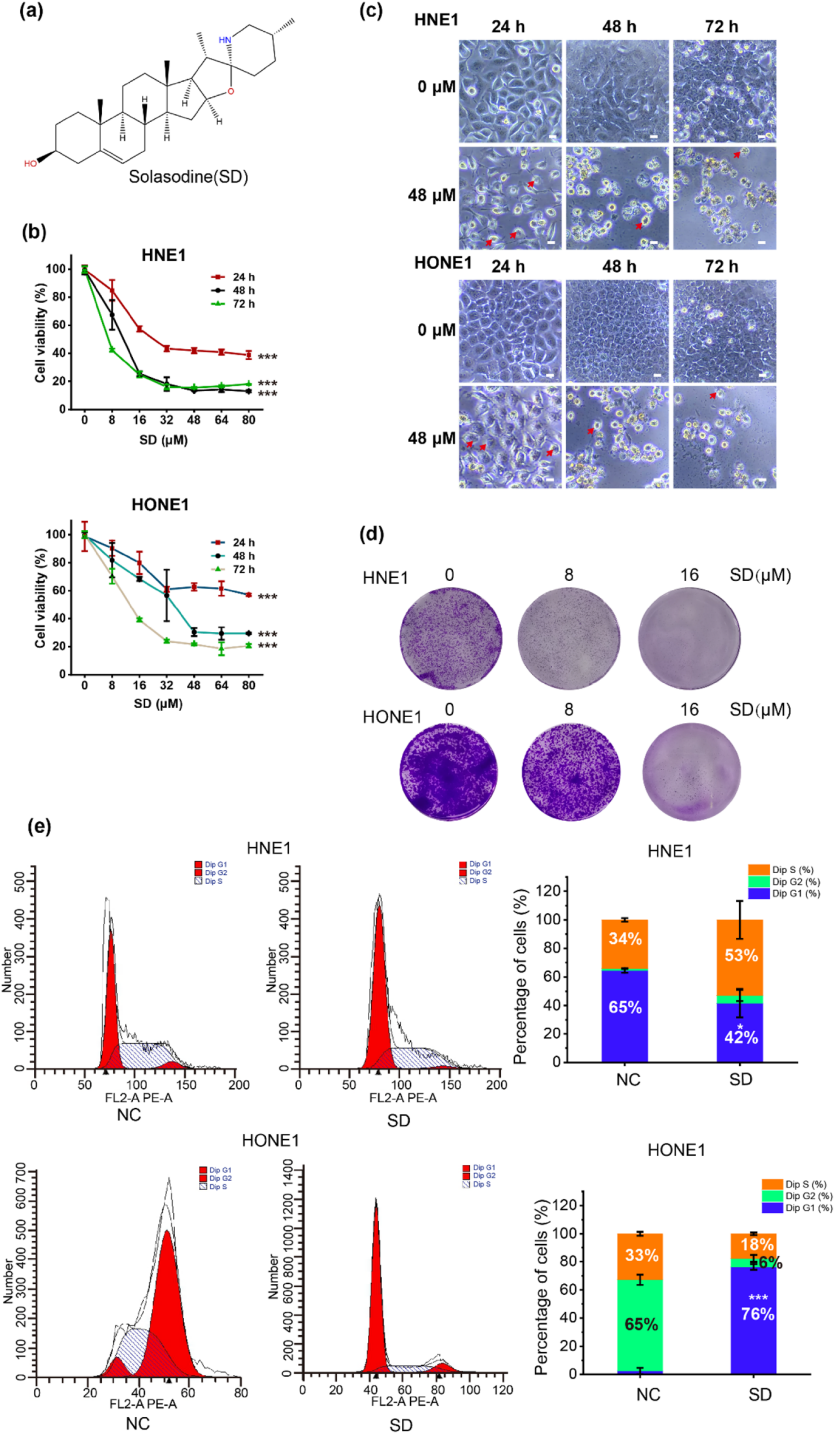
The cytotoxicity of the solasodine in NPC cells. (**a**) Molecular structures of solasodine. (**b**) Cell viability was assessed by CCK-8 assay. (**c**) Morphology observation of NPC cells after treatment with 48 μM of solasodine for 24, 48, and 72 h. The red arrow marks the cells with a balloon-like phenotype. Scale bar: 10 μm. (**d**) The inhibition ability of solasodine to the colony forming of NPC cells after treatment with 8 and 16 μM of solasodine. (**e**) Cell cycle was determined by flow cytometry 24 h after treatment with 48 μM of solasodine. Top left: HNE1, bottom left: HONE1. Quantitative analysis of cells in each cell cycle phase in NPC cells. Top right: HNE1, bottom right: HONE1. Mean values and standard deviation were calculated from three independent experiments, n = 3. *, **, and *** indicate statistically significant differences with *p* < 0.05, 0.01 and 0.001, respectively.

To explore the cytotoxic effects of solasodine (see Fig. 1a for its chemical structure) on NPC cell lines HNE1 and HONE1, we treated these cells with escalating concentrations of solasodine (0, 8, 16, 32, 48, 64, 80 µM) for 24, 48, and 72 h, and assessed cell viability using the CCK8 assay. The results indicated that solasodine inhibited NPC cells’ viability in a time and concentration-dependent manner (Fig. 1b). The IC_50_ of solasodine were 33.51 µM (24 h), 12.81 µM (48 h) and 3.35 µM (72 h) for HNE1 cells, and 115.6 µM (24 h), 34.74 µM (48 h) and 13.65 µM (72 h) for HONE1 cells. Furthermore, after treatment with 48 µM of solasodine, cell morphology changed significantly, such as distinct ballooning features and cell swelling (Fig. 1c). The colony formation assay confirmed that solasodine reduced the clonal formation of HNE1 and HONE1 cells in a dose-dependent manner, demonstrating its antiproliferative activity (Fig. 1d). Additionally, solasodine also affected DNA replication of NPC cells. Treatment with 48 µM of solasodine induced cell cycle arrest at G0/G1 phase (DNA pre-synthesis phase) compared to the control group in HONE1 cells, but induced cell cycle arrest at S phase (DNA synthesis stage) compared to the control group in HNE1 cells (Fig. 1e). Overall, the results from viability assays, morphology observation, colony formation assay, and cell cycle assay indicated that solasodine possesses potent cytotoxicity against NPC cells.

### Solasodine promotes NPC cell death

**Fig. 2:**
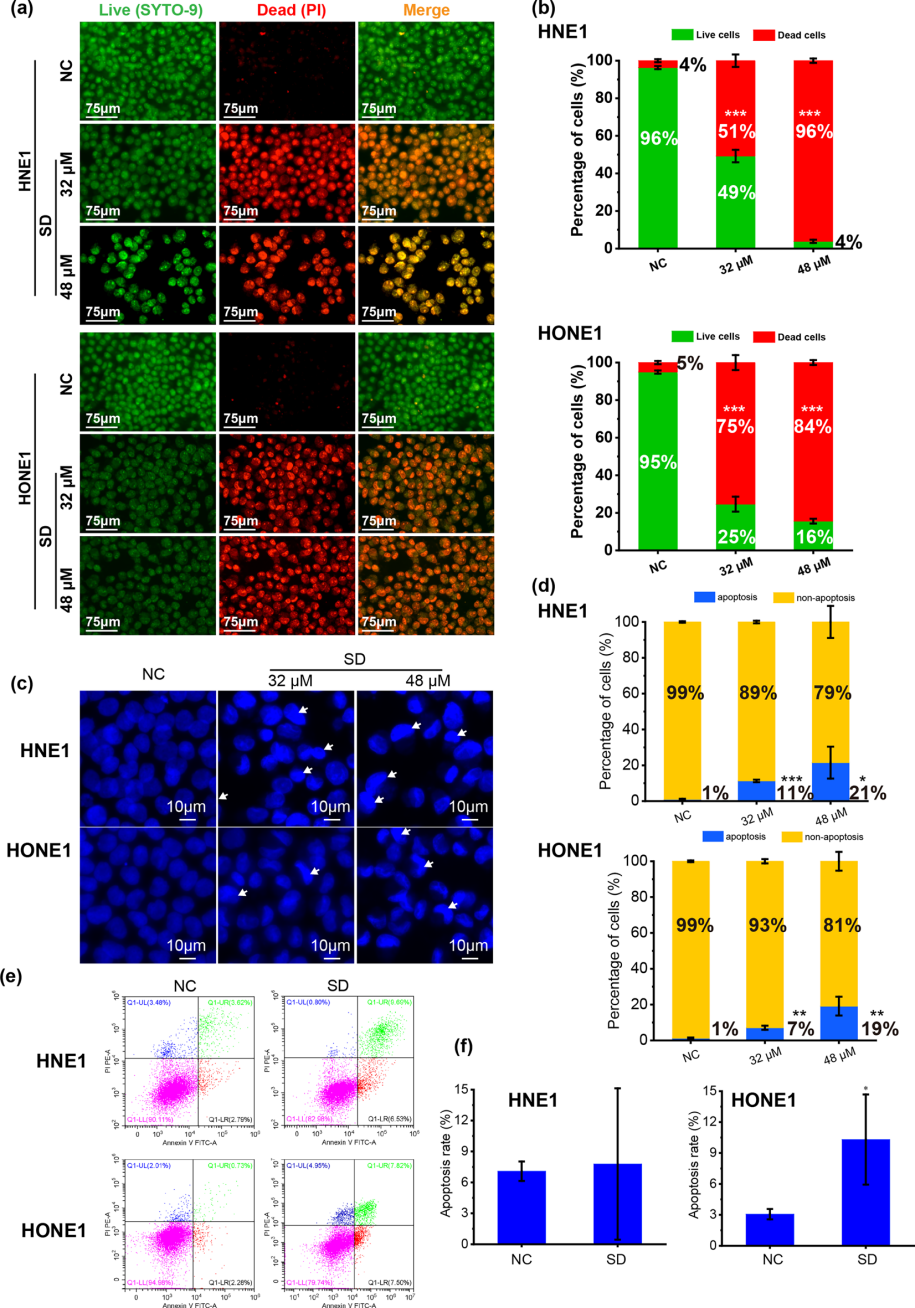
Solasodine promotes NPC cells death. (**a**) Confocal micrographs of HNE1 and HONE1 cells labeled with SYTO-9 (green represented the live cells) and propidium iodide (red indicated the dead cells) after treatment with 32 and 48 μM of solasodine for 24 h. Scale bars: 75 μm. (**b**) Percentage of live and dead cells on different treatment groups. (**c**)The apoptosis of NPC cells treated with 32 and 48 μM of solasodine for 24 h were measured by Hoechst staining. (**d**) Quantitative analysis of the apoptotic percentage of NPC cells measured by Hoechst staining. (**e**) Representative picture of Annexin V-FITC cell apoptosis assay in 32 μM of solasodine treatment group or control group. (**f**) Quantitative analysis of the apoptotic percentage of NPC cells measured by Annexin V-FITC cell apoptosis assay. Bars represent the mean ± standard deviation of three independent experiments, n = 3. *, **, and *** indicate statistically significant differences with *p* < 0.05, 0.01 and 0.001, respectively.

In order to visually observe the survival/death status of NPC cells, the cells were treated with 32 and 48 µM of solasodine for 24 h, and then stained with a live/dead staining (SYTO-9 and propidium iodide) for imaging. As shown in Fig. 2a,b, a large number of green livings HNE1 and HONE1 cells appeared in the control group, while the 32 and 48 µM of solasodine treatment groups showed relatively more red dead cells. The percentage of living cells and dead cells in the control group and the treatment group were further statistically analyzed. As shown in Fig. 2b, the percentage of live cells for HNE1 cells after treatment with 32 and 48 µM of solasodine were 49% and 4%, which was significantly lower than the control group (96%) (*p* < 0.001). Similar to HNE1, the percentage of live cells for HONE1 cells after treatment with 32 and 48 µM of solasodine were 25% and 16% compared to 95% of control group (*p* < 0.001). Hoechst staining results showed cellular nuclear shrinkage in solasodine treated cells, with nuclei appearing bright blue and crescent-shaped. ImageJ analysis indicated a marked increase in the proportion of apoptotic cells after treatment with 32 and 48 µM of solasodine (Fig. 2c,d). Similarly, the Annexin V-FITC cell apoptosis assay results showed that the control group exhibited minimal damage or early apoptotic cells, whereas 32 µM of solasodine treatment led to a significantly higher cell apoptosis rate of HNE1 and HONE1 at (7.79 ± 7.34)% (ns) and (10.30 ± 4.37)% (*p* < 0.05), respectively (Fig. 2e,f). These findings collectively illustrate the capacity of solasodine to induce NPC cell death.

### Solasodine inhibits tumor growth in xenograft nude mice

**Fig. 3:**
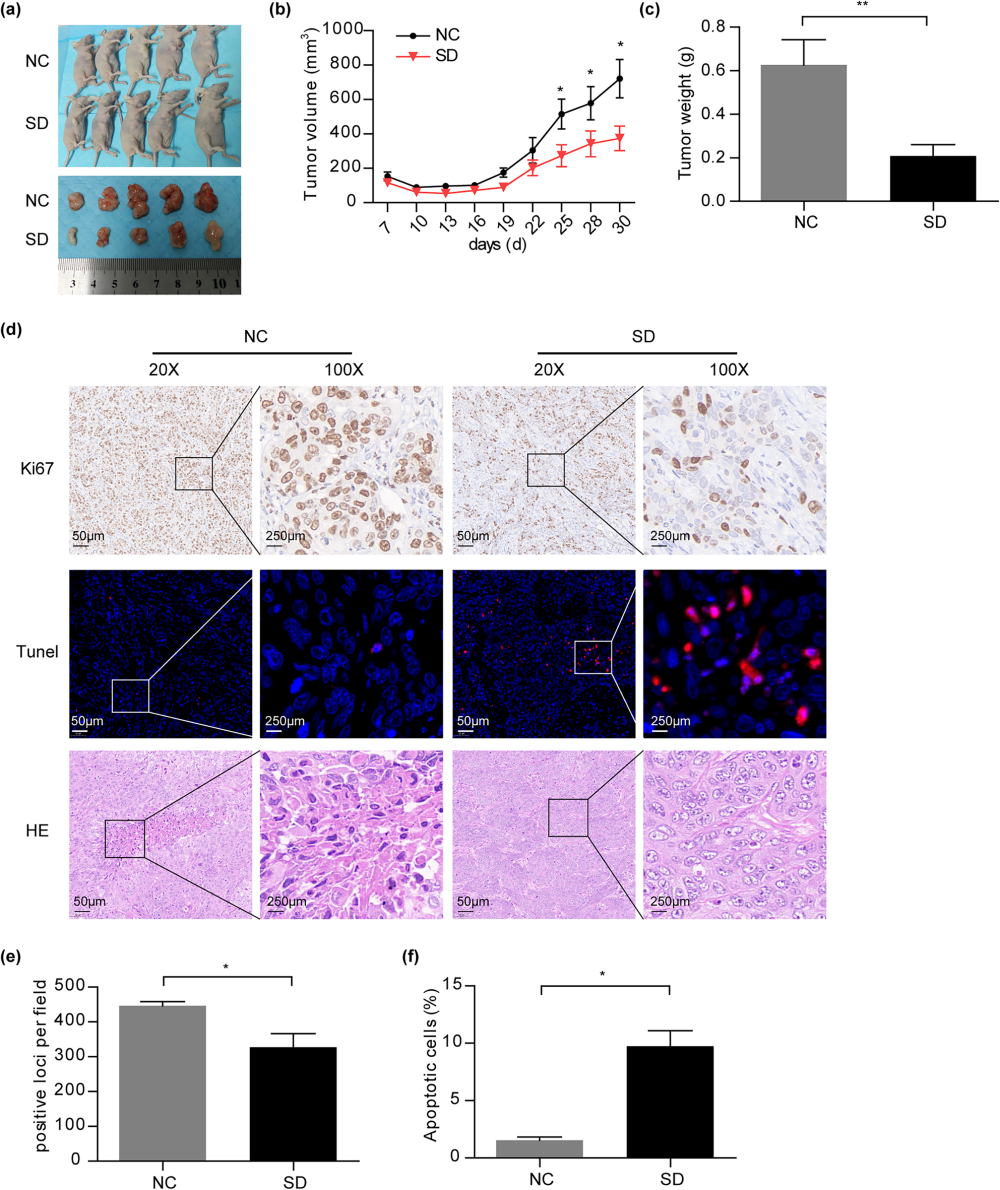
Solasodine inhibits xenograft tumor growth of HNE1 cells in BALB/c female nude mice. (**a**) The typical tumor-bearing mice and isolated xenograft tumors from PBS group (NC) and solasodine group (SD). (**b**) Tumor volume changes in each treatment group (n = 5). (**c**) Tumor weight changes in each treatment group after dissection (n = 5). (**d**) Histological analysis of tumor tissues from NC and SD groups. The paraffin-embedded histological sections of tumors were stained with anti-Ki-67 antibody (up), TUNEL staining (middle), and hematoxylin and eosin (H&E, down). (**e**) Quantitative analysis of Ki-67 expression. (**f**) Percentage of apoptotic cells in tumor tissue. *, **, and *** indicate statistically significant differences with *p* < 0.05, 0.01 and 0.001, respectively.

To assess the antitumor efficacy and safety of solasodine in vivo, NPC xenograft model was developed in BALB/c female nude mice using HNE1 cells. Once tumor size reached approximately 100 mm^3^, animals were treated with vehicle control (PBS) or solasodine (SD, 10 mg/kg dissolved in methanol) intraperitoneally every 3 days for 3 weeks. Treatment with solasodine significantly inhibited tumor growth compared to the PBS group (tumor volumes: 591.1 ± 50.38 mm^3^ vs. 1430 ± 413 mm^3^) (Fig. 3a,b). The tumor weight was significantly lower in solasodine group compared to the PBS group (2.716 ± 0.2557 g vs. 3.636 ± 0.5569 g) (Fig. 3c). Moreover, histological examination revealed decreased nuclear content and enlarged interstitial spaces in the solasodine group, which was distinct from the heteromorphic appearance in the PBS group (Fig. 3d). Immunohistochemical analysis showed the expression of Ki-67 was significantly lower in solasodine treated tumor tissues than that in PBS group (Fig. 3d,e). The TUNEL assay further confirmed increased apoptosis in the solasodine group, with a higher number of tumor cells displaying red fluorescence (Fig. 3d,f). Safety assessments indicated that solasodine treatment did not result in significant weight loss nor histopathological alterations in major organs such as the kidney and liver (Fig. S1), affirming the effective yet non-toxic nature of solasodine in suppressing xenograft tumor growth.

### Network pharmacology analysis reveals that ferroptosis participates in solasodine-induced NPC cell death

**Fig. 4:**
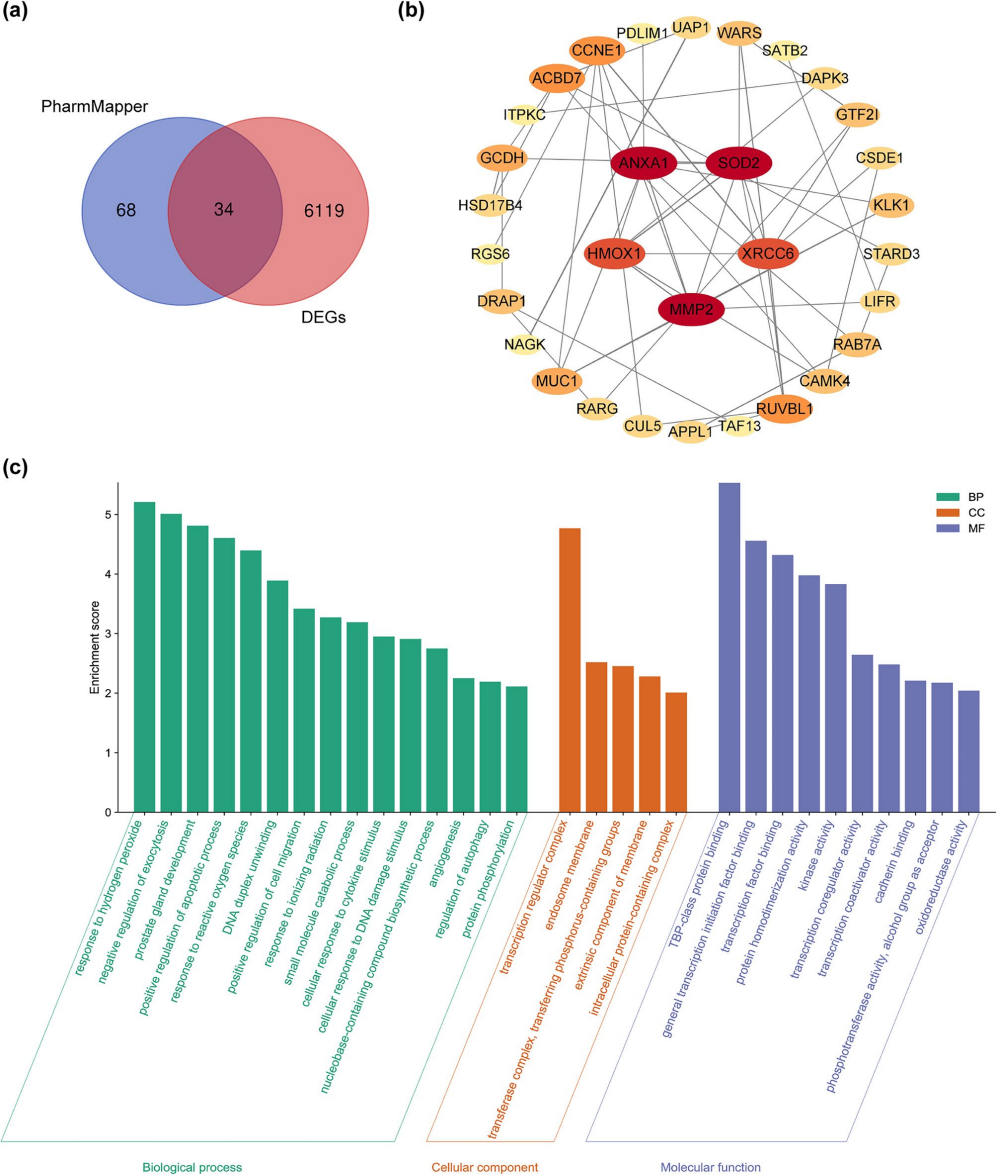
Network pharmacology analysis reveals the target for solasodine-induced cell death in NPC cells. (**a**) Venn intersection diagram of solasodine target in PharmMapperr database and RNA-seq data of differential expressed genes after treatment with solasodine. (**b**) PPI network analysis and core gene analysis of 34 shared target genes. (**c**) GO functional annotation enrichment analysis of 34 shared target genes.

Given the anti-NPC cell effects exhibited by solasodine in vitro and in vivo, we aimed to uncover its potential mechanisms of action. We searched the PharmMapper database for proteins matching the pharmacophore models of solasodine in humans and obtained 102 target proteins. By intersecting the predicated solasodine targets with DEGs of NPC cells treated with solasodine, we obtained 34 shared targets using a Venn diagram (Fig. 4a). These 34 targets were uploaded to the STRING platform to construct a protein–protein interaction (PPI) network, followed by core gene analysis using Cytoscape, leading to the identification of the top 5 core target genes: ANXA1, SOD2, HMOX1, MMP2, and XRCC6 (Fig. 4b, Fig. S2). Notably, HMOX1—a pivotal gene associated with the iron-dependent cell death pathway known as ferroptosis—stood out as a critical target, implying that solasodine’s anti-NPC activity could be mediated by triggering ferroptosis. Enrichment analysis of Gene Ontology (GO) terms for the 34 core genes highlighted their roles in biological processes like responding to reactive oxygen species (ROS) and hydrogen peroxide, as well as positively regulating the apoptotic process. Cellular component analysis revealed these genes’ localization within the endosome membrane, while molecular function analysis indicated their oxidoreductase activity (Fig. 4c). These findings align with the functional role of the core target gene HMOX1 (Fig. S3), reinforcing the potential link between solasodine and ferroptosis in NPC cells.

### Solasodine increases ROS generation and disrupts mitochondrial morphology

**Fig. 5:**
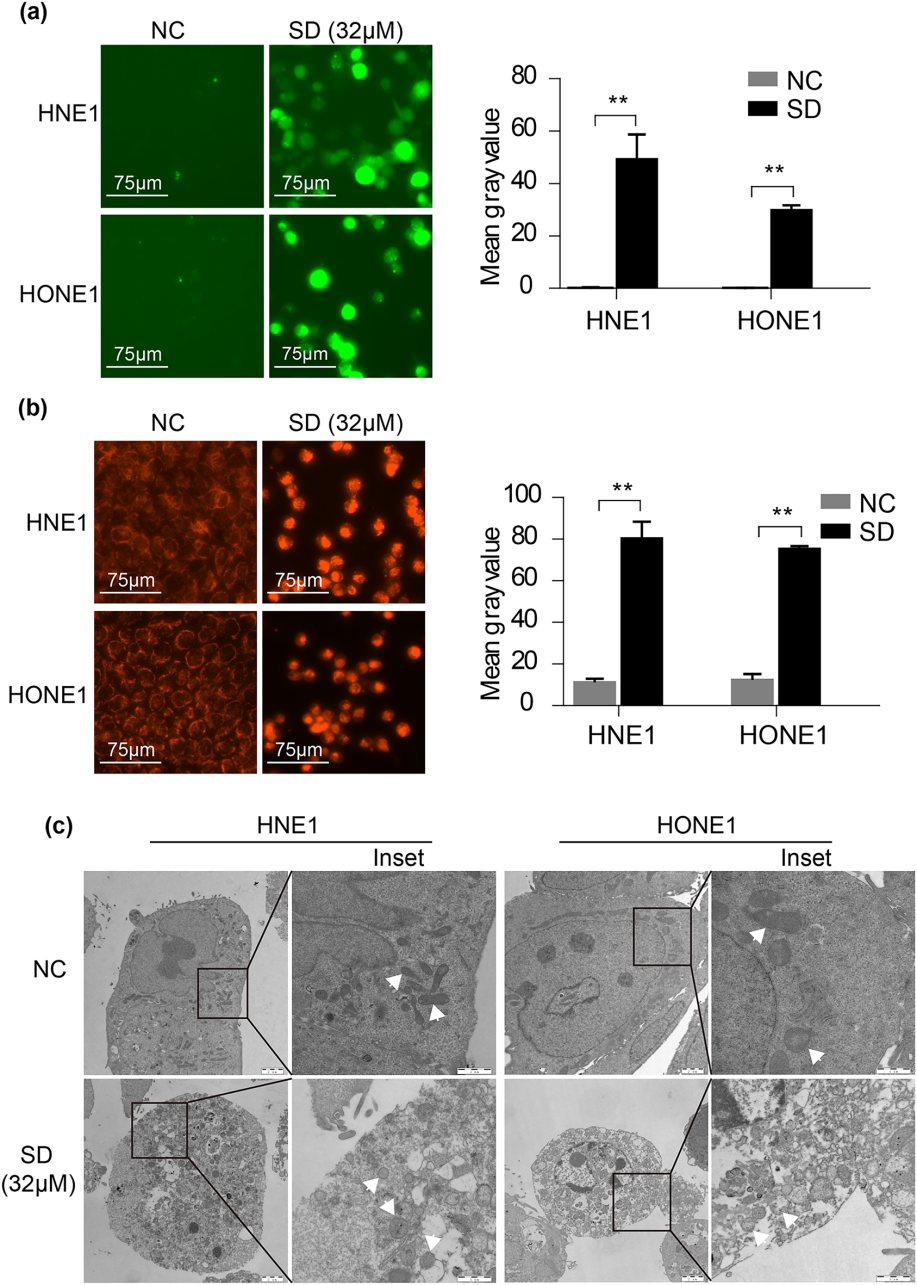
Solasodine increased ROS generation and destroyed mitochondrial morphology in NPC cells. (**a**) Solasodine at concentrations of 32 µM increased intracellular ROS levels in NPC cells. Left: Typical green fluorescence imaging of ROS. Right: Quantitative analysis of the fluorescence intensity of ROS by DCFH-DA staining. (**b**) Mitochondrial morphological accumulation in MitoTracker Red CMXRos-stained NPC treated with 32 µM of solasodine for 24 h. Left: Typical fluorescence imaging of mitochondrial morphological changes. Right: Quantitative analysis of the mitochondrial fluorescence intensity. (**c**) TEM was used to observe mitochondrial morphology of NPC induced by solasodine (32 µM). Inset: The partial enlargement of TEM images of typical mitochondrial morphology (white arrows). Scale bars: 1 and 2 µm. Bars represent the mean ± standard deviation of three independent experiments, n = 3. **indicates statistically significant differences with *p* < 0.01.

Cellular ROS mainly originates from mitochondria. Our investigation utilizing the ROS assay kit (DCFH-DA) revealed that after 24-h exposure with 32 µM of solasodine, the cells exhibited markedly brighter and significantly elevated green fluorescence indicative of ROS production (Fig. 5a left, right). Additionally, Mito-Tracker Red CMXRos staining demonstrated a shift from the diffuse red fluorescence pattern in the control group to a pronounced aggregation of red fluorescence in solasodine-treated cells, indicating alterations in mitochondrial integrity (Fig. 5b left, right). Transmission electron microscopy (TEM) further corroborated these findings. While mitochondria in the control group maintained their typical ellipsoid shape with intact cristae, those in the solasodine group displayed a range of aberrant morphologies, including hollow centers, round shapes, and disrupted or diminished mitochondrial cristae (Fig. 5c; Fig. S4). These observations align with the hallmarks of mitochondrial changes of ferroptosis. Integrating these results with the functional analysis of the core target gene HMOX1 derived from our network pharmacology study, we proposed that ferroptosis could play a role in the cytotoxic effects of solasodine on NPC cells.

### Solasodine-induced NPC cell death involves ferroptosis

**Fig. 6:**
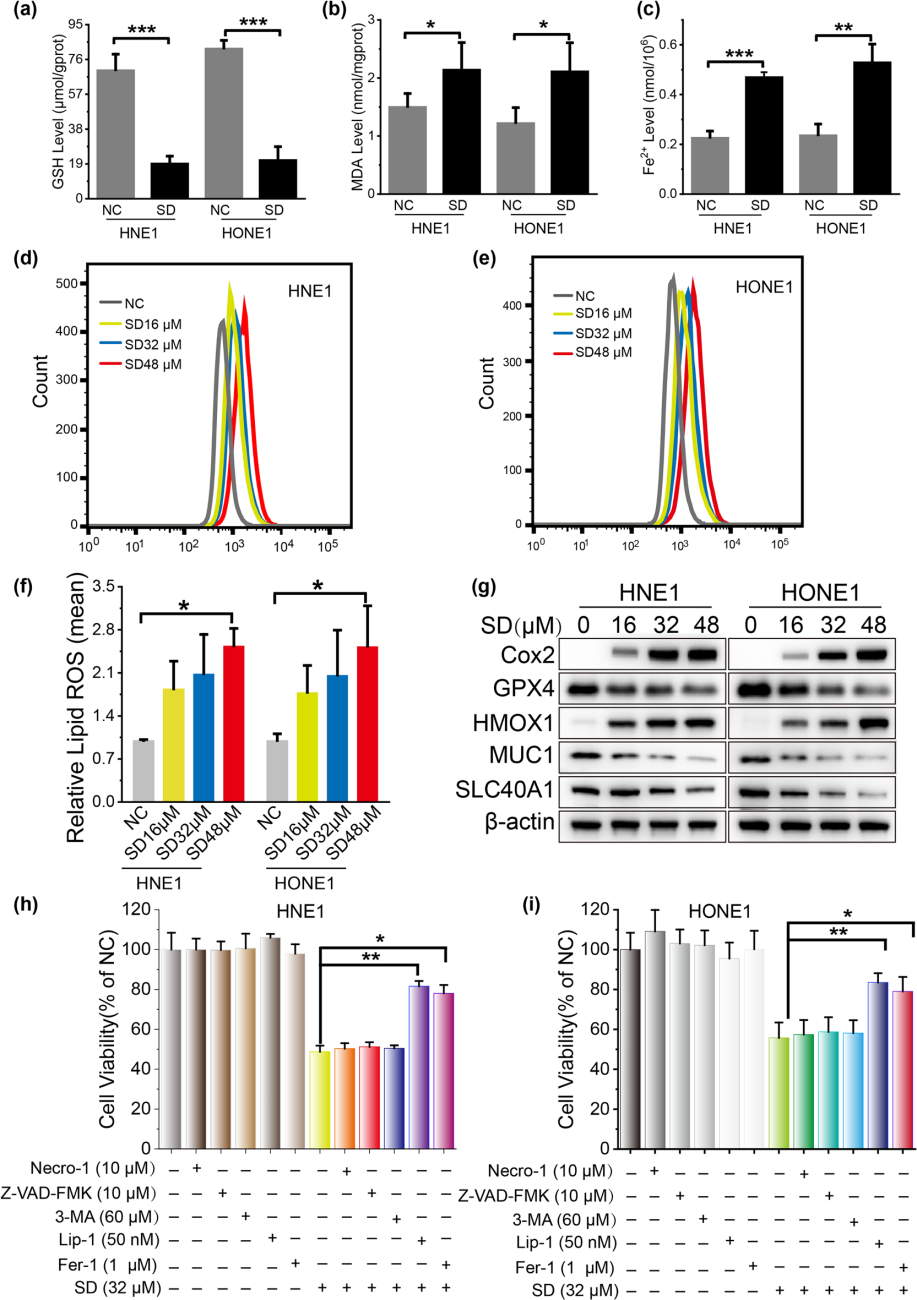
Ferroptosis plays a role in solasodine-induced cell death in NPC cells. Intracellular GSH (**a**), MDA (**b**), and Fe^2+^ levels (**c**) in NPC cells treated with solasodine. The lipid ROS level of HNE1 (**d**) and HONE1 (**e**) analyzed by a flow cytometer in NPC cells treated with solasodine (16, 32, 48 µM). (**f**) Quantitative analysis of mean fluorescent intensity of the lipid ROS. (**g**) The expression of several key ferroptosis regulators was examined by western blotting. (**h**, **i**) The cell viability of HNE1 (**h**) and HONE1 (**i**) was assayed after treatment with the necrosis inhibitor (Necro-1), apoptosis inhibitor (Z-VAD-FMK), autophagy inhibitor (3-MA), and ferroptosis inhibitor liproxstatin-1 (Lip-1) or ferrostatin-1 (Fer-1) alone or in co-incubation with solasodine. Bars represent the mean ± standard deviation of three independent experiments, n = 3. *, **, and *** indicate statistically significant differences with *p* < 0.05, 0.01 and 0.001, respectively.

To validate the role of ferroptosis and oxidative stress in solasodine-induced NPC cell death, we assessed key indicators of redox homeostasis. Consistent with our hypothesis, we observed a substantial reduction in glutathione (GSH) levels and a concomitant elevation in malondialdehyde (MDA) subsequent to solasodine treatment (Fig. 6a,b). Intracellular levels of free Fe^2+^ ions and lipid-associated reactive oxygen species (ROS) were also notably increased in solasodine-treated NPC cells, indicating ferroptosis initiation (Fig. 6c–f). Additionally, the expression of heme oxygenase-1 (HMOX1) and cyclooxygenase-2 (Cox2) were upregulated in a dose-dependent manner following solasodine exposure, while the expression of ferroptosis suppressors, including glutathione peroxidase 4 (GPX4), mucin 1 (MUC1), and solute carrier family 40 member 1 (SLC40A1), were downregulated (Fig. 6g). Furthermore, co-incubation of solasodine with necrosis inhibitor (Necro-1), apoptosis inhibitor (Z-VAD-FMK), and autophagy inhibitor (3-MA) did not alter the cell viability of NPC cells, but co-treatment of solasodine with ferroptosis inhibitors liproxstatin-1 (Lip-1) or ferrostatin-1 (Fer-1) reversed solasodine-induced cell death (Fig. 6h,i). Taken together, these comprehensive findings strongly suggest that solasodine elicits ferroptotic cell death in NPC cells.

## Discussion

In the face of increasingly prominent obstacles such as drug resistance and side effects in cancer chemotherapy, developing more effective and safer alternative therapies is urgent. Among numerous anticancer strategies, natural compounds are garnering attention due to their distinctive bioactive structures, potential multi-target mechanisms, and lower toxic side effects. The abundant variety of nature has given rise to numerous medicinal compounds with significant anticancer properties, which may directly trigger cancer cell death, inhibit cancer cell migration, or modulate the tumor microenvironment, showcasing a range of anticancer mechanisms^27–30^.

This research delves into exploring the potential worth of solasodine (SD) in the treatment of NPC. SD is a natural compound derived from the traditional herb *Solanum nigrum L.*. Previous studies have confirmed that SD possesses a wide array of biological activities, especially its anticancer potential. SD can effectively inhibit the progression of various tumors such as colorectal cancer, breast cancer, gastric cancer, ovarian cancer, and lung cancer by impeding cell proliferation, inducing cell apoptosis, regulating cell stemness, and suppressing cell invasion and migration^12–15,31^. However, the exploration of SD in the context of NPC remains limited.

Our findings demonstrate that SD significantly impedes the growth and viability of NPC cells and promotes cell death in vitro and in vivo. The IC50 values of solasodine in HNE1 cells were 33.51 µM (24 h), 12.81 µM (48 h), and 3.35 µM (72 h), while in HONE1 cells they were 115.6 µM (24 h), 34.74 µM (48 h), and 13.65 µM (72 h). These values are within a similar range compared to previous studies in other cancer models. For instance, in colorectal cancer cells (48 h, IC50: 39.43–50.09 μM)^12^, breast cancer cells (72 h, IC50: 2.86–28.80 μM)^13^, and ovarian cancer cells (24 h, IC50: 34.05–55.27 μM; 48 h, IC50: 23.08–29.34 μM)^15^. These comparisons indicate that SD exhibits potent anticancer activity across different cancer types, with NPC cells showing a similar sensitivity profile to other cancer cells. To further clarify the underlying mechanism of SD’s anticancer effect against NPC, we utilized a network pharmacology approach to systematically analyze SD’s potential targets and molecular mechanisms in NPC treatment. We identified 5 hub targets of SD in NPC, including ANXA1, SOD2, HMOX1, MMP2, XRCC6. Among them, HMOX1 caught our attention for its critical role in ferroptosis regulation. This suggests that SD may achieve its anticancer effects by targeting HMOX1 and interfering with the ferroptosis pathway.

Recently, ferroptosis, as a novel mode of cell death. Recently, ferroptosis has garnered widespread attention as a novel mode of cell death, particularly in cancer research. Unlike traditional apoptosis and necrosis, the core mechanism of ferroptosis involves excessive accumulation of intracellular iron ions and uncontrolled lipid peroxidation. This process is regulated by various molecules and signaling pathways^32–36^, including iron homeostasis regulators such as ferritin and hepcidin that regulate the uptake, storage, and release of iron within cells; lipid peroxidation-related enzymes such as lipoxygenases (LOXs) and cyclooxygenases (COXs) that participate in lipid metabolism and the generation of peroxides; and antioxidant defense systems such as glutathione peroxidase (GPX4) and thioredoxin (TRX) that clear intracellular peroxides and protect cells from oxidative damage. Ferroptosis can act as an effective mechanism to limit the proliferation and spread of tumor cells, while cancer cells may also enhance their antioxidant defenses to resist ferroptosis, conferring a survival advantage.

Although research on ferroptosis in NPC is still insufficient, there are indications that ferroptosis assumes a pivotal role in the onset and progression of NPC^37–39^. Since SD is a hydrolysis product of solasonine, and the latter has been proven to induce ferroptosis in hepatocellular carcinoma, pancreatic cancer, and lung adenocarcinoma cells^40–42^, we speculate that SD may induce NPC cell death through the ferroptosis pathway. Fortunately, subsequent experiments by detecting the typical hallmarks of ferroptosis confirmed this hypothesis. Through a series of in vitro experiments, we observed significant morphological changes in the mitochondria of NPC cells after treatment with SD. These changes included a reduction in size, and the decrease or disappearance of mitochondrial cristae. Additionally, there was a notable increase in the levels of intracellular ferrous iron ions (Fe^2+^), reactive oxygen species (ROS), and lipid peroxidation, along with a decrease in the levels of glutathione (GSH). These alterations are highly consistent with the typical characteristics of ferroptosis. Moreover, the targeted application of ferroptosis inhibitors Fer-1 and Lip-1, both of which are widely utilized in the field of ferroptosis research and have been extensively documented for their specificity in inhibiting ferroptosis^43,44^, partially reversed cell death, in contrast to the use of inhibitors for other cell death modalities such as Z-VAD-FMK for apoptosis, Necro-1 for necroptosis, and 3-MA for autophagy. This finding highlights the novel mechanism of SD against NPC by inducing ferroptosis.

In our study, we observed significant changes of key proteins involved in the ferroptosis pathway after SD treatment, such as GPX4, HMOX1, COX2, SLC40A1, and MUC1, which jointly promoting the occurrence of ferroptosis in NPC cells. While HMOX1 is typically associated with protective responses against oxidative stress, its upregulation in this context may contribute to ferroptosis by increasing the labile iron pool, which is consistent with previous research indicating that HMOX1 can exacerbate lipid peroxidation by releasing iron^45,46^. In terms of iron export, ferroportin (SLC40A1), once known as the sole iron efflux pump that cooperates with ceruloplasmin or hephaestin, plays a crucial role in cellular iron efflux. The downregulation of SLC40A1 leads to iron accumulation within the cell and exacerbating ferroptosis^47^. Cyclooxygenase 2 (Cox2), a key enzyme in inflammatory responses, may contribute to lipid peroxidation through the production of prostaglandin H2 (PGH2), and its increased expression in this study could further amplify oxidative damage during ferroptosis^48^. Concurrently, the downregulation of negative regulators such as glutathione peroxidase 4 (GPX4) and mucin 1 (MUC1) may weaken the cell’s antioxidant defenses, making it more susceptible to oxidative stress. GPX4 is a key regulator of ferroptosis, and its dysfunction or downregulation will lead to the accumulation of lipid peroxides. Downregulation of MUC1 may affect the stability of the cell membrane and the function of the cystine/glutamate transporter (system XC-), thereby promoting ferroptosis^49,50^. These molecular alterations provide mechanistic insights into the anticancer effects of SD and suggest that targeting ferroptosis could be a promising approach for the treatment of NPC.

Our research provides new insights into the role of SD as an anticancer drug and its induction of ferroptosis in NPC, differing from previous studies. Firstly, while prior research has demonstrated the anticancer effects of SD in various cancers^12–15^, our work specifically investigates its role in NPC, a disease with unique pathological features and limited treatment options. Secondly, we conducted a comprehensive analysis of the molecular mechanisms of SD using network pharmacology, identifying key targets such as ANXA1, SOD2, HMOX1, MMP2, and XRCC6 that have not been previously reported in the context of NPC. SD may exert multiple anticancer effects by targeting these molecules, such as inhibiting tumor cell growth and invasion, regulating tumor cell stemness, modulating tumor immunity, and suppressing angiogenesis^51–55^. Thirdly, our data suggest that targeting ferroptosis with SD is a novel therapeutic strategy for NPC, a finding that not only highlights new therapeutic targets for NPC but also enhances the understanding of SD’s anticancer potential and opens new avenues for its application in the treatment of nasopharyngeal carcinoma.

Despite the promising results presented in this study, several limitations must be acknowledged. First, our findings are primarily based on in vitro experiments and in vivo mouse models, and the anticancer effects of SD remain to be validated in more complex models, such as patient-derived xenograft (PDX) models or clinical trials. These studies will provide important insights into the efficacy and safety of SD for human patients. Second, although the identification of key targets such as ANXA1, SOD2, HMOX1, MMP2, and XRCC6 using network pharmacology, the exact regulatory pathways involved in SD-induced ferroptosis in NPC cells remain unclear and require further clarification. While network pharmacology provides a powerful tool for identifying potential molecular targets, it also has inherent limitations. Computational predictions generated by network pharmacology may include false positives due to incomplete databases, algorithmic biases, or insufficient biological context. Therefore, experimental validation is necessary to confirm the relevance and accuracy of these predicted targets. Third, while our data strongly suggest that ferroptosis is the main mechanism of SD-induced cell death in NPC cells, the fact that ferroptosis inhibitors failed to completely reverse cell death indicates potential crosstalk with other cell death pathways. A growing body of research indicates that crosstalk exists between different modes of cell death^56,57^. In particular, increasing evidence suggests that ferroptosis may interact with autophagy, necroptosis, or metabolic stress in a context-dependent manner. For instance, autophagy can enhance intracellular iron levels by selectively degrading ferritin (iron storage protein), thereby promoting ferroptosis^58^, while lipid peroxidation-a hallmark feature of ferroptosis-may synergize with membrane damage caused by necroptosis^59^. Notably, our inhibitor experiments ruled out apoptosis, necrosis, or autophagy as sole contributors but do not exclude their auxiliary role in regulating ferroptosis signaling. Future research could further dissect these interactions by exploring time-dependent pathway activation or gene silencing of key regulatory factors such as GPX4 and RIPK1. Additionally, the long-term effects of SD treatment on normal tissues and potential off-target effects have yet to be fully assessed. While our study demonstrates that SD effectively induces ferroptosis in NPC cells, it is crucial to acknowledge the potential for off-target effects, especially at the high concentrations used in our experiments. Natural compounds like SD often exhibit complex pharmacological profiles due to their multi-target nature, which can lead to unintended impacts on non-cancer cells or unrelated pathways. Future research should focus on optimizing the concentration and administration of SD to minimize potential off-target effects while maintaining its therapeutic efficacy. Furthermore, the pharmacokinetic properties of SD, including its bioavailability and tissue distribution, require detailed investigation. While our in vivo experiments demonstrated that SD significantly inhibits tumor growth in xenograft nude mice, the specific mechanisms underlying its biodistribution and metabolic fate remain unclear. For instance, the ability of SD to reach and accumulate in the tumor site could influence its therapeutic efficacy. Future studies should employ advanced techniques, such as mass spectrometry or imaging technologies, to comprehensively analyze the pharmacokinetics of SD in preclinical models. Addressing these gaps is crucial for translating our findings into clinical practice.

The translational implications of our research findings is promising but also presents significant challenges. The discovery of SD-induced ferroptosis in NPC cells offers a potential new therapeutic strategy for this aggressive cancer. However, translating these findings into clinical practice will require overcoming several obstacles. First, the bioavailability and pharmacokinetics of SD need to be optimized to ensure effective delivery to the tumor site. Second, potential off-target effects and toxicity must be thoroughly investigated in preclinical and clinical studies. Furthermore, developing SD as a therapeutic agent also necessitates exploring its synergistic effects with existing therapies, such as chemotherapy or immunotherapy, to enhance treatment efficacy. Future research should also focus on identifying biomarkers to predict patients’ responses to SD treatment, enabling more personalized and effective cancer therapies. Additionally, large-scale clinical trials will be needed to confirm the therapeutic benefits of SD in human patients, providing critical data on its safety, efficacy, and overall impact on patient outcomes.

**Fig. 7:**
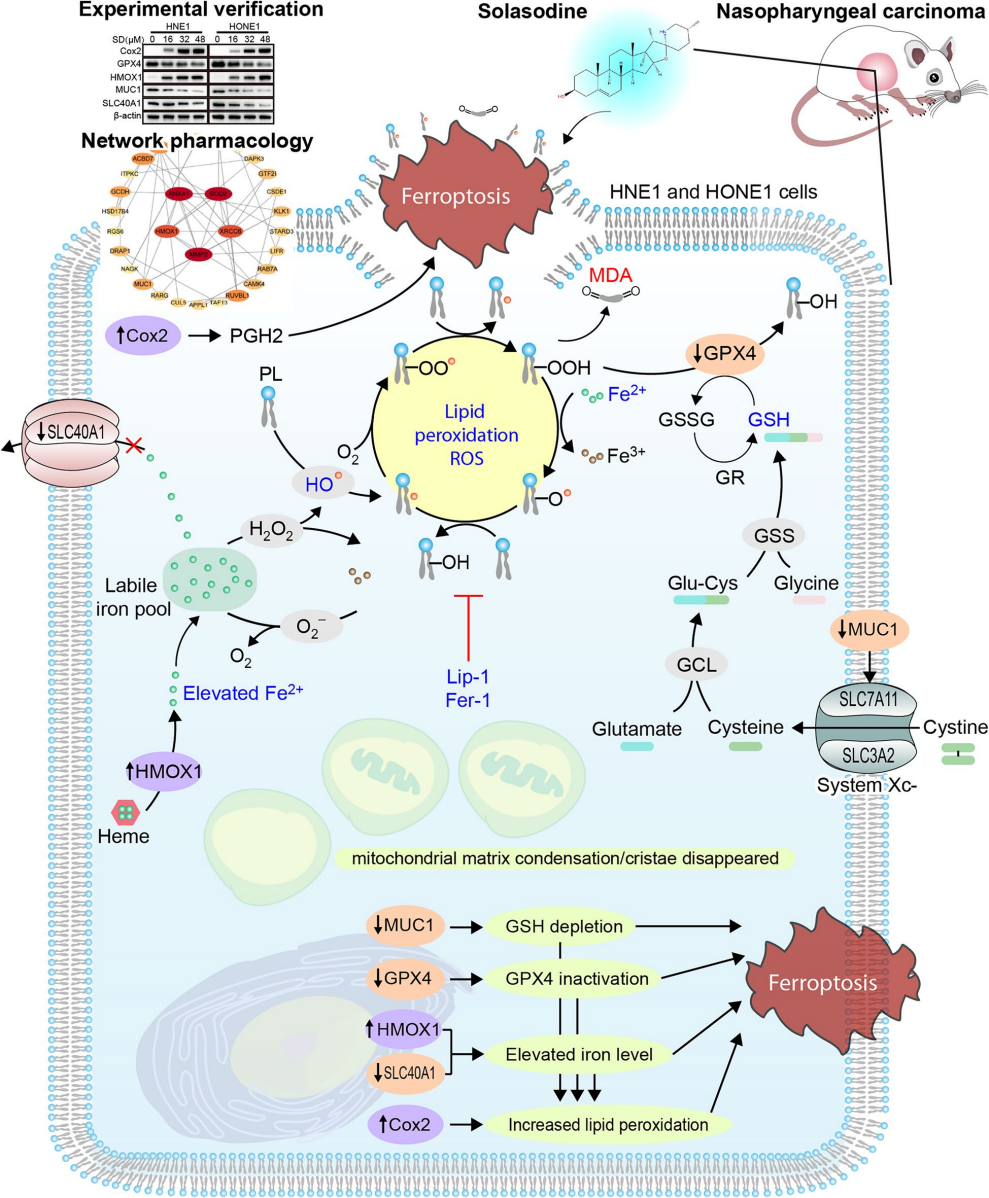
The potential mechanism of solasodine inhibiting the progress of NPC.

In conclusion, our findings demonstrate that SD disrupts the redox balance in NPC cells, leading to a significant increase in intracellular iron levels and lipid peroxidation, ultimately triggering ferroptosis (Fig. 7). This discovery not only offers a novel perspective on the anticancer effects of SD but also highlights the potential of targeting ferroptosis as a new therapeutic strategy for NPC. By elucidating SD’s role in modulating the ferroptosis pathway, our study underscores the importance of exploring non-apoptotic cell death mechanisms in cancer treatment. Our research shows that SD can effectively induce ferroptosis by targeting key regulators such as HMOX1, thereby inhibiting tumor growth and promoting cell death. This finding suggests that ferroptosis may serve as an effective mechanism to overcome the limitations of traditional apoptosis-based therapies, which often face challenges such as drug resistance and side effects. Targeting ferroptosis could provide a more effective and safer alternative to conventional chemotherapy, particularly for cancers like NPC that have limited treatment options. By leveraging the multi-target nature of natural compounds like SD, we can develop therapies that not only induce cell death through ferroptosis but also modulate the tumor microenvironment, inhibit tumor cell migration, and enhance overall treatment efficacy. Future research should focus on optimizing the pharmacokinetic properties of SD, exploring its synergistic potential with existing therapies, and identifying biomarkers to predict patient responses. These efforts will be crucial for translating our findings into clinical practice. In summary, our study provides a strong foundation for advancing SD as a promising candidate for NPC treatment. By addressing the limitations identified and exploring the potential of SD in combination with other therapies, we can pave the way for more effective and personalized cancer treatments, ultimately improving patient outcomes and quality of life.

## Supplementary Information

Supplementary Information.

Supplementary Figures.

## Data Availability

The data used to support the findings of this study are included within the article and supplementary file.
