# Supplementary material for: Solasodine suppresses nasopharyngeal carcinoma progression by inducing ferroptosis
Source: Sci Rep. 2025 May 18;15:17247. doi: 10.1038/s41598-025-93834-4 (PMC12086188; doi:10.1038/s41598-025-93834-4)

HNE1 COX2

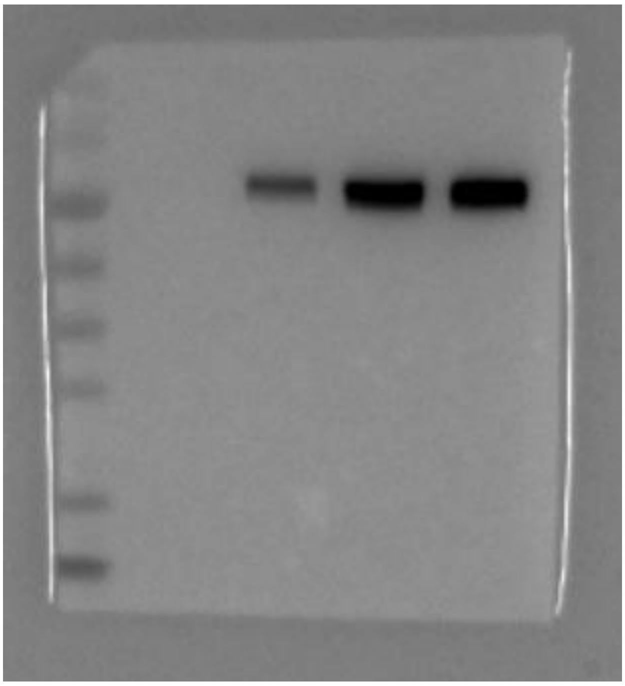

HNE1 GPX4

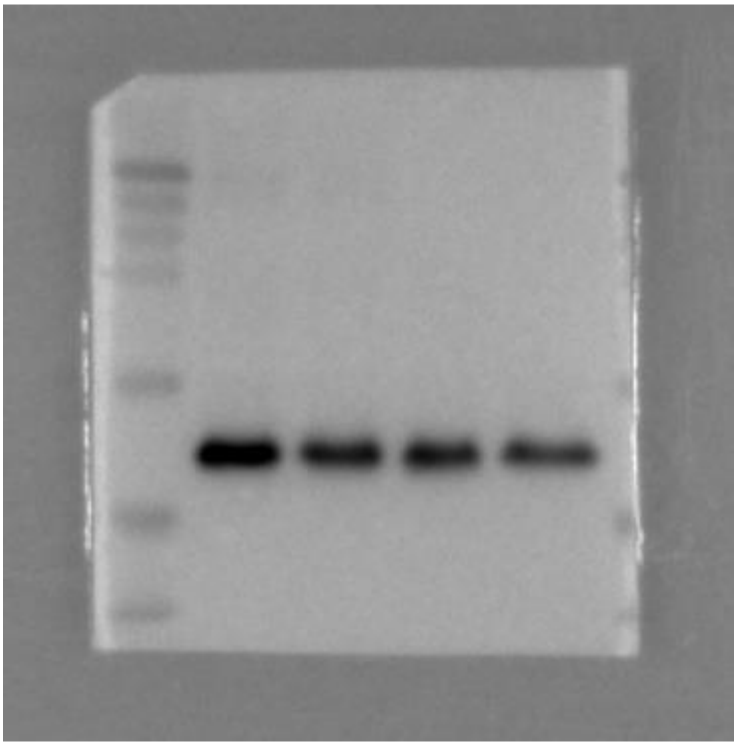

HNE1 HMOX1

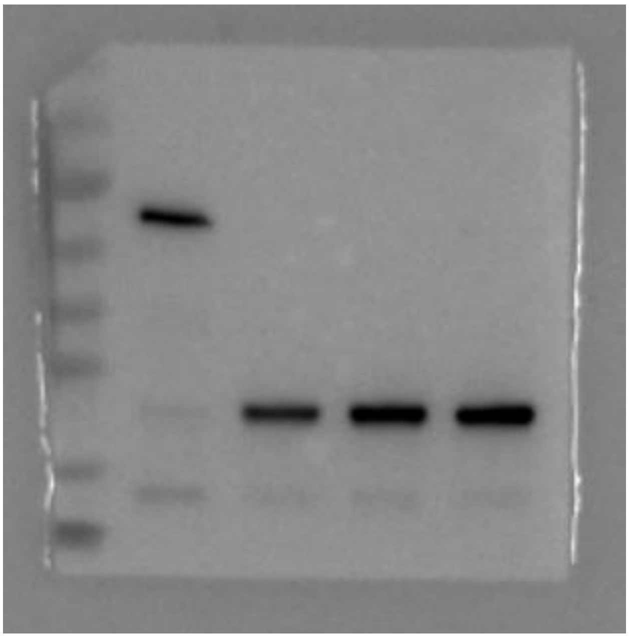

HNE1 MUC1

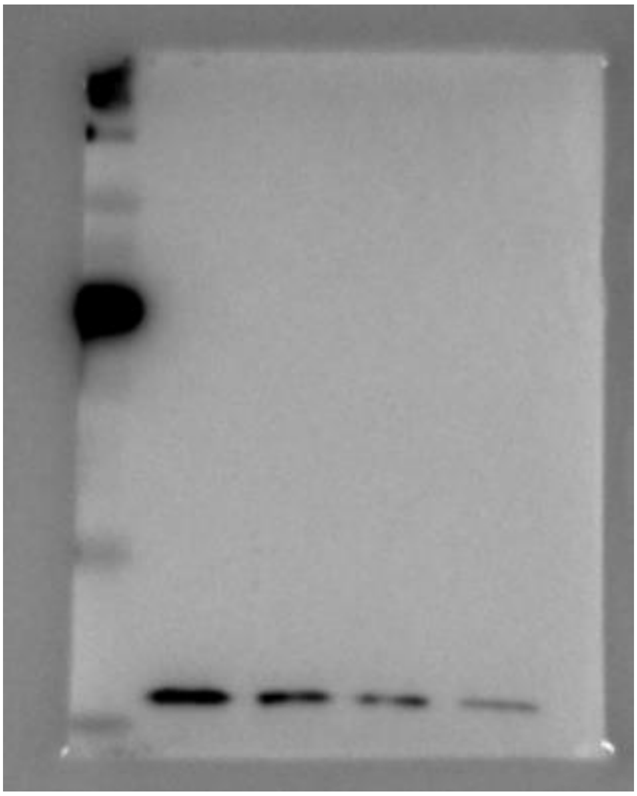

HNE1 SLC40A1

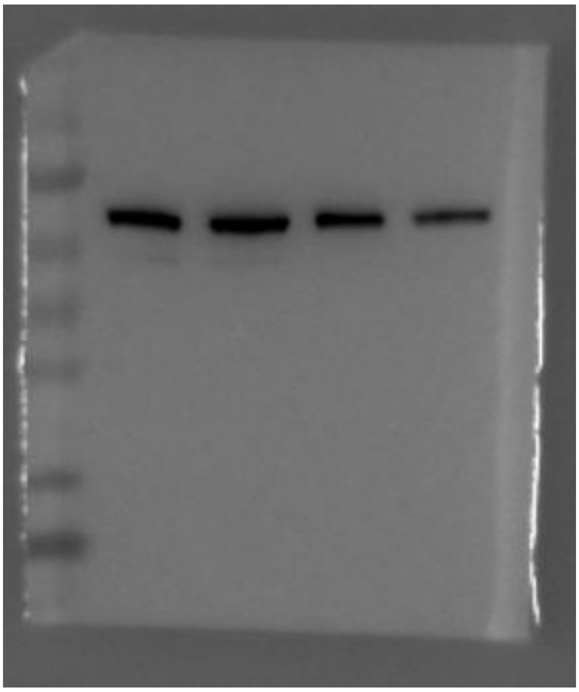

HNE1 actin

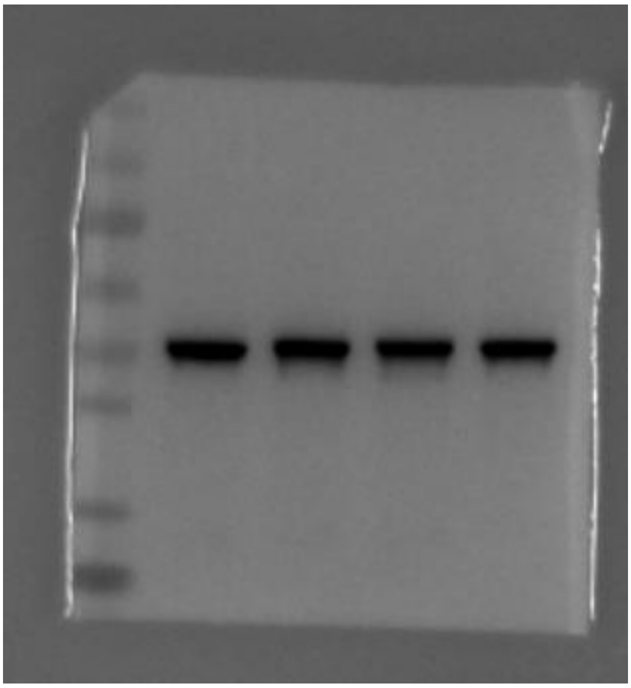

HONE1 COX2

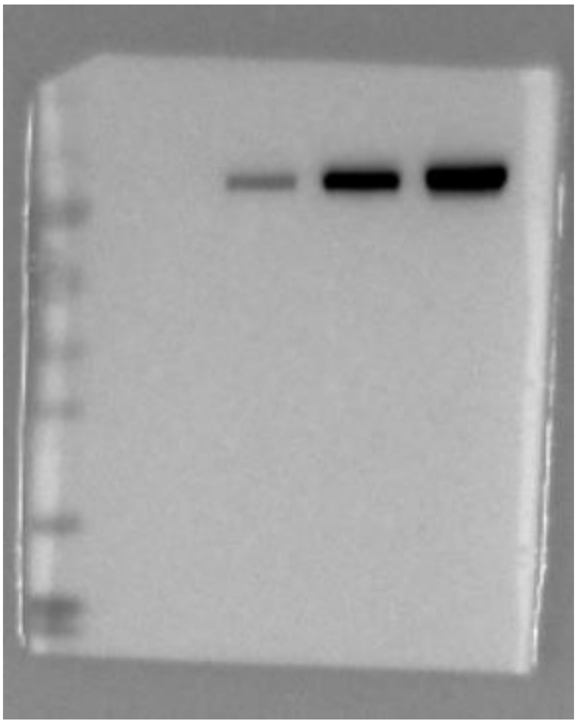

HONE1 GPX4

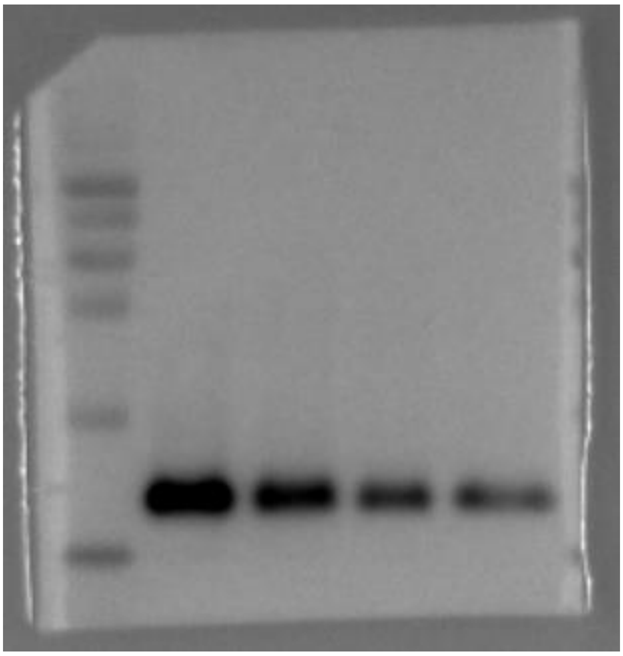

HONE1 HO1

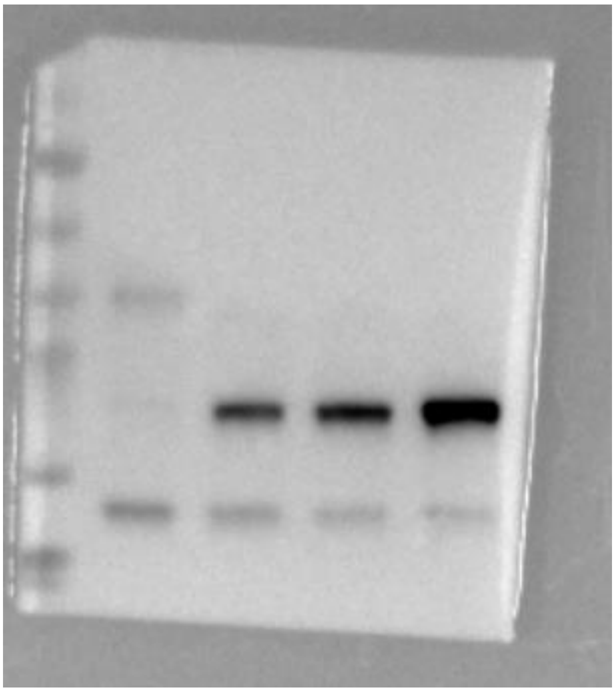

HNE1 MUC1

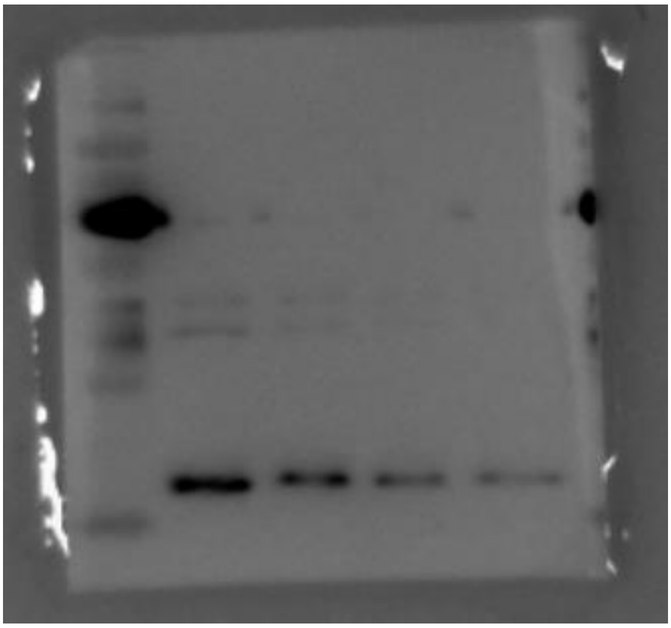

HONE1 SLC40A1

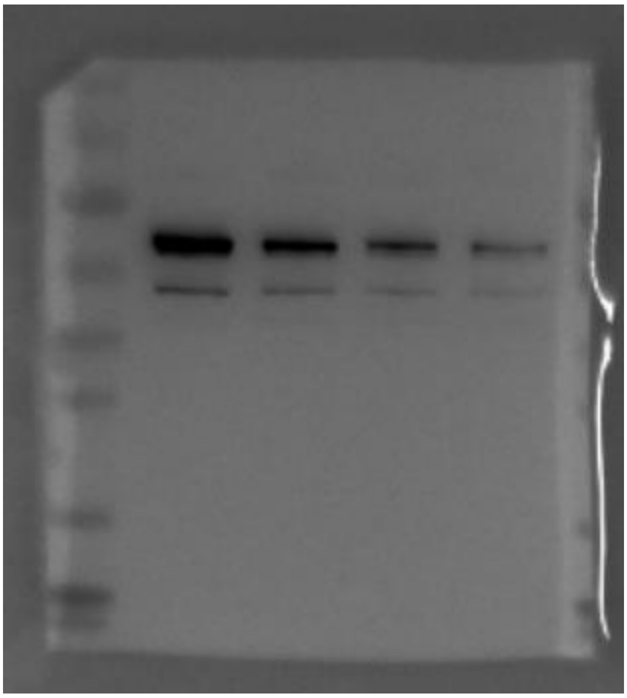

HONE1 actin

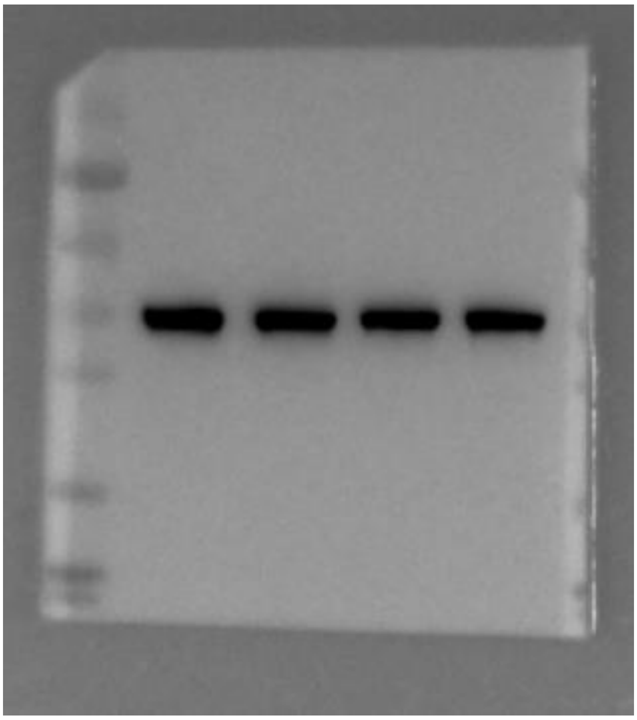

Supplement: Supplementary file 1 — Supplementary Information. [file 41598_2025_93834_MOESM1_ESM.pdf]
