# Supplementary material for: Solasodine suppresses nasopharyngeal carcinoma progression by inducing ferroptosis
Source: Sci Rep. 2025 May 18;15:17247. doi: 10.1038/s41598-025-93834-4 (PMC12086188; doi:10.1038/s41598-025-93834-4)
Supplement: Supplementary file 2 — Supplementary Figures. [file 41598_2025_93834_MOESM2_ESM.docx]

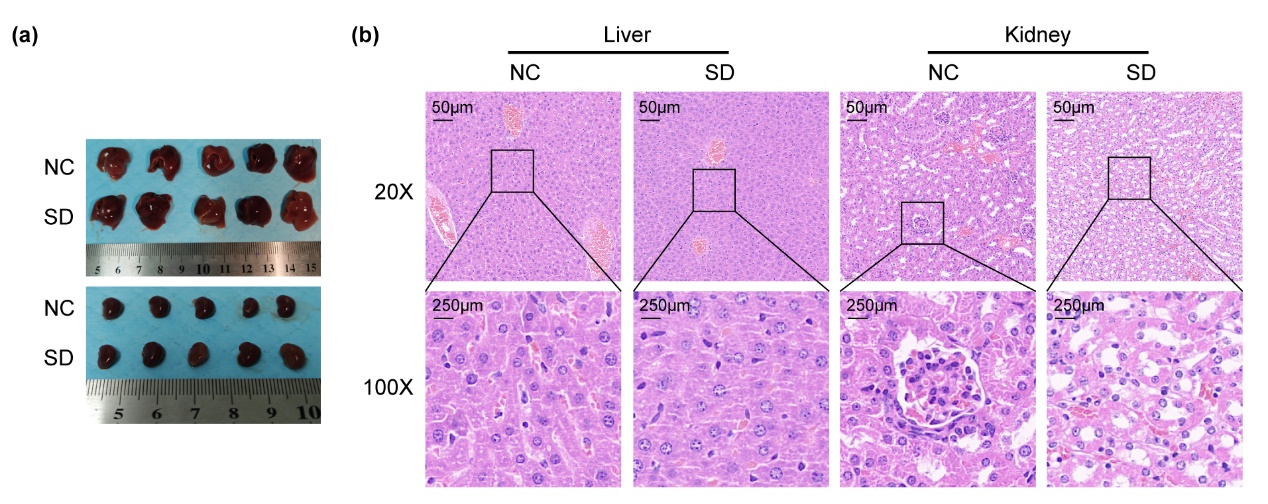


**Supplementary Figure 1.** **The safety of solasodine was evaluated in vivo by observing the changes in vital organs in mice.**

(a) The size of the liver and kidney were observed after dissection. (b) Cell morphology in liver and kidney of mice from groups treated with SD and PBS were analyzed by hematoxylin and eosin (H&E) staining. Scale bar: 50 μm and 250 μm.


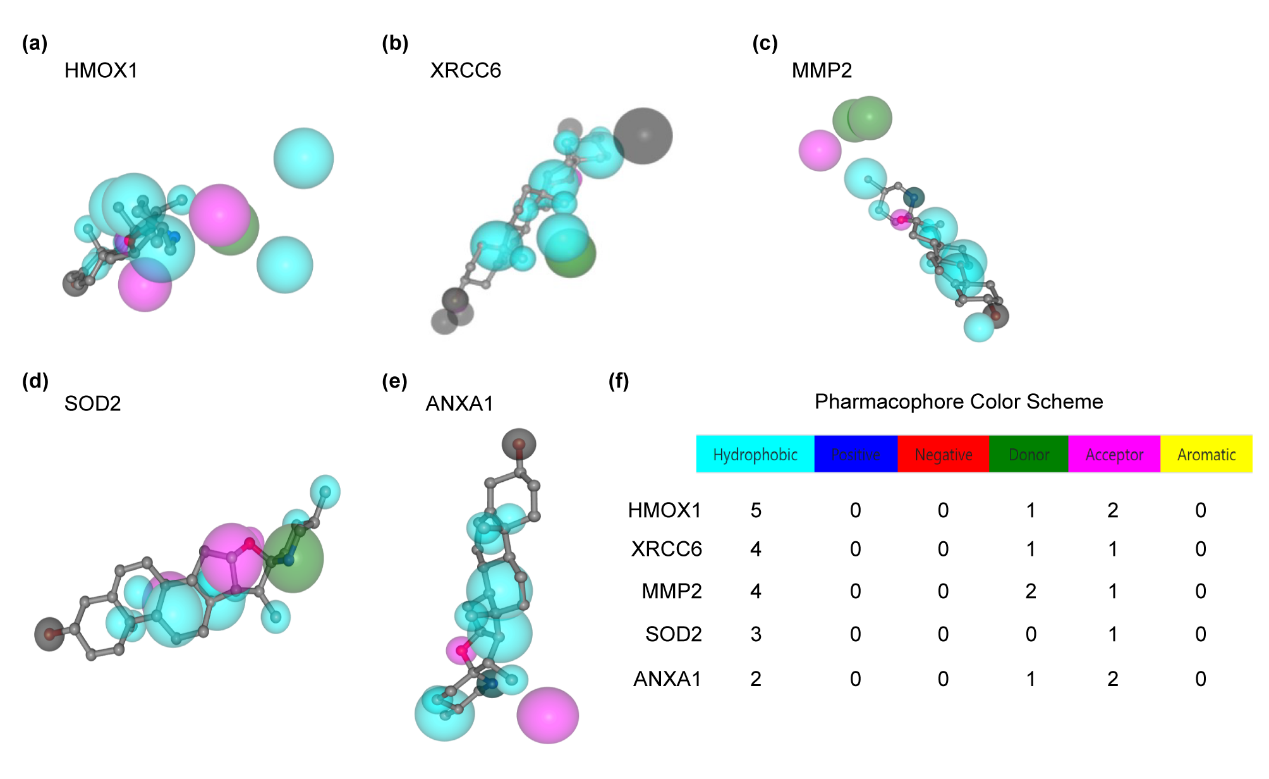


**Supplementary Figure 2. Binding site prediction between Solasodine and the five core target genes.**

HMOX1 (a), XRCC6 (b), MMP2 (c), SOD2 (d), and ANXA1 (e). (f) The pharmacophore feature of the five core targets.


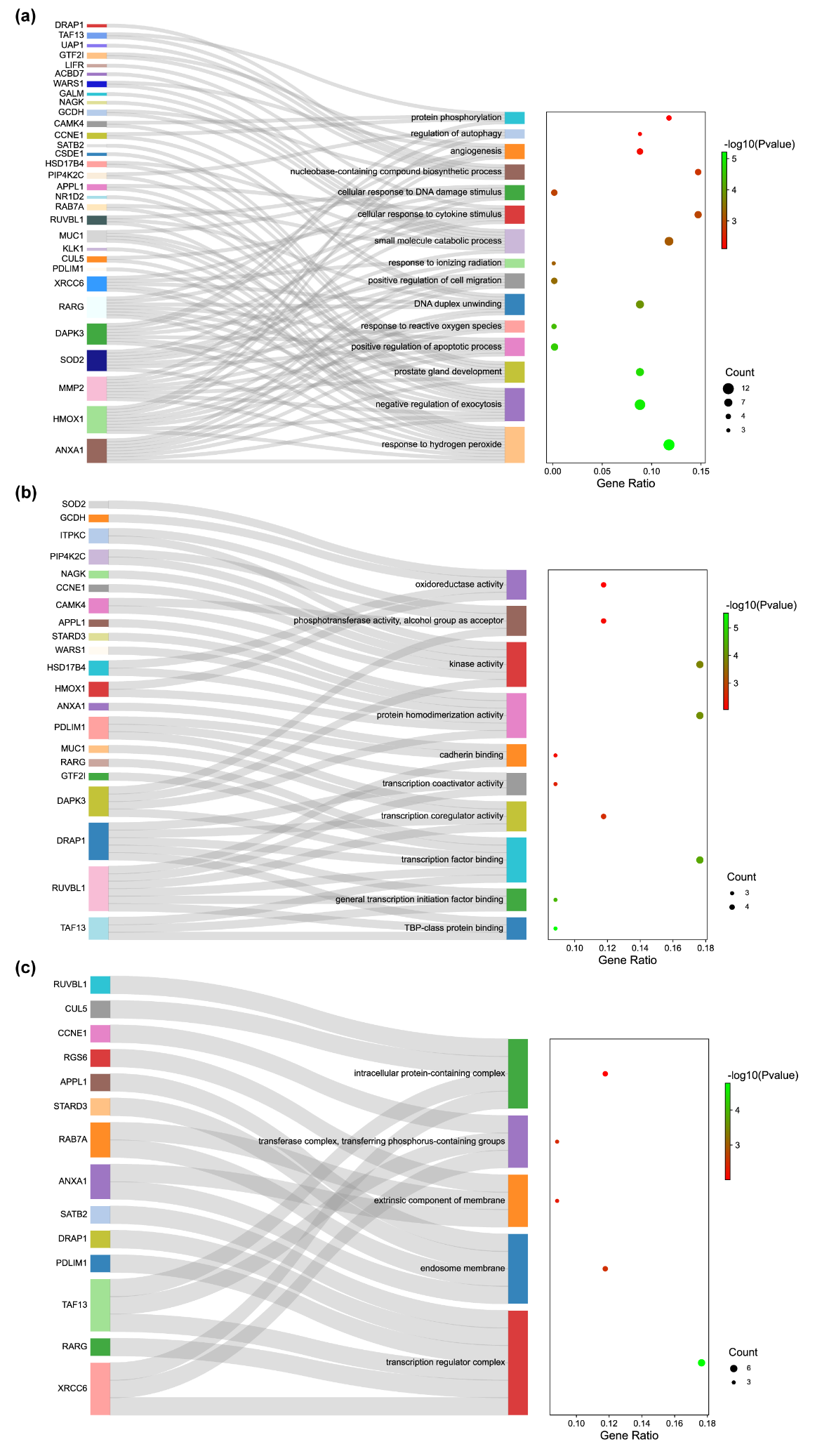


**Supplementary Figure 3. GO enrichment analysis of the 34 target genes obtained from the intersection of the SD target genes and DEGs.**

1. GO-biological process analysis. (b) GO-molecular function analysis. (c) GO-cellular component analysis.


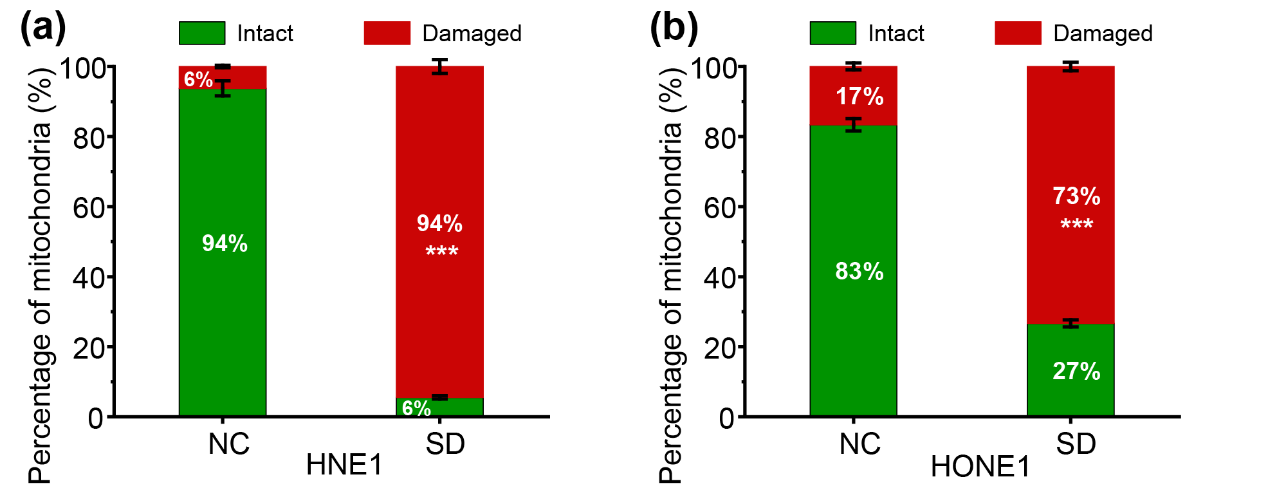


**Supplementary Figure 4. Effect of solasodine on mitochondrial damage of NPC cells.**

1. Percentage of mitochondria was analyzed based on TEM results in HNE1 cells. (b) Percentage of mitochondria was analyzed based on TEM results in HONE1 cells.
